# Creating and sustaining collaborative multi-institutional industry site visit programs: a toolkit

**DOI:** 10.12688/f1000research.26598.2

**Published:** 2022-10-28

**Authors:** Tammy R.L. Collins, Kiri Hoff, Molly Starback, Patrick D. Brandt, Christopher E. Holmquist, Rebekah L. Layton

**Affiliations:** 1 Office of Fellows' Career Development, National Institute of Environmental Health Sciences, USA, Research Triangle Park, North Carolina, 27709, USA; 2 Genome Integrity and Structural Biology Laboratory, National Institute of Environmental Health Sciences, USA, Research Triangle Park, North Carolina, 27709, USA; 3 Office of Postdoctoral Services, Duke University, Durham, North Carolina, 27710, USA; 4 Office of Graduate Education, University of North Carolina at Chapel Hill, Chapel Hill, North Carolina, 27599, USA; 5 Biochemistry Department, U.T. Southwestern Medical Center, Dallas, TX, 75390, USA

**Keywords:** graduate and postdoctoral professional development, experiential learning, industry site visit program, biomedical workforce, career outcomes

## Abstract

**Background:** As more early career scientists enter into diverse career pathways, visiting local companies or organizations can support their exploration of these paths. As an efficient way to facilitate this, we developed a collaborative regional site visit program: the Enhancing Local Industry Transitions through Exploration (ELITE) Consortium.  Consortium members arrange half-day visits to local industry sites, thus providing companies and trainees the opportunity to meet and identify potential professional and career opportunities. Three different training institutions worked cooperatively in the development and maintenance of the program. The ELITE Consortium was developed with eight phased steps; guidelines and operating procedures were created for each of these steps and are provided along with sample materials for institutions interested in building similar programs.

**Methods: **Prior to fully developing the program, trainee interests were evaluated via questionnaire. During program implementation and thereafter, program directors tracked attendance and collected career outcome data from publicly available sources to identify first job positions after training. Regression analyses and chi-squared analyses were used to examine site visit matches and career outcome data.

**Results: **Analyses suggest a positive impact of site visits on postdoctoral and graduate trainees’ career outcomes at companies or institutions that match a similar sector (e.g., for-profit) and type (e.g., biotech, pharmaceutical, contract research organization). Despite a small sample size, evidence suggests an especially positive impact on trainees who organize site visits to companies compared with those who simply participate.

**Conclusions: **The ELITE Consortium was successful in helping trainees explore and identify a multitude of career paths. Trainees attained employment either directly or in related companies and institutions visited by ELITE participants. The joint, three-institution, flexible nature of the ELITE Consortium positively impacts the program’s sustainability and reach. The toolkit provided here will help other institutions to replicate and adapt the program with minimal effort.

## Introduction

It is widely known that the number of tenure-track positions remains relatively flat while the number of PhD-holders increases ( National Institutes of Health [NIH], 2012)—meaning many individuals will enter into other types of careers beyond faculty positions ( Stayart *et al.,* 2020). Therefore, institutions should be preparing their graduate students and postdoctoral scholars for these other types of careers ( Sinche *et al.,* 2017)—and this idea finally seems to be gaining traction in academe ( Lenzi *et al.,* 2020). Preparing students and postdocs for such careers can take on many forms, and one example is through company site visits. While this has long been common practice in the professional degree-seeking communities and for undergraduate students (e.g., business, engineering; see Velez & Giner, 2015 and Carbone *et al.,* 2020 for reviews), PhD-level trainees in the scientific research training community (particularly in STEM and the biosciences) have had limited applications of this learning model until recently.

To address this gap in career preparation, experiential learning has recently been applied more broadly in graduate education ( Schnoes *et al.,* 2018; Van Wart *et al.,* 2020). The NIH Broadening Experiences in Scientific Training (BEST) Consortium has deployed career development training across four major types of experiential learning: job simulation, site visits, job shadowing, and internships ( Van Wart *et al.,* 2020). Each of these variations of experiential learning activities has varying levels of engagement and skill acquisition, but also can require very intensive resource and time commitments (e.g., high dose of experiential learning potential in resource-intensive internship). However, not all trainees are at a place in their professional development where they are ready to invest the time and resources needed to take advantage of these intensive experiences; furthermore, not all institutions have resources or staff available to coordinate such time-intensive options. Fortunately, lower-dose and less time-intensive options may also provide benefits to trainee-participants. The current work explores the effectiveness of delivering career development experiential learning though site visits organized across a multi-institutional collaboration, creating an efficient method to deliver potentially valuable professional development experiences.

The focus of this manuscript is a company site visit program termed the Enhancing Local Industry Transitions through Exploration (ELITE) Consortium. The mission of the ELITE Consortium is to connect companies that hire PhDs with PhD students and postdocs from the National Institute of Environmental Health Sciences (NIEHS), the University of North Carolina: Chapel Hill (UNC), and Duke University (Duke). ELITE helps trainees explore industry career options through site visits to leading Research Triangle Park, NC, life-science companies, and to other employers beyond the traditional tenure-track (e.g., University-operated contract research organizations, ( https://factor.niehs.nih.gov/2016/11/science-highlights/elite/index.htm). Site visits allow trainees to learn about the different types of industry jobs open to PhDs and how best to prepare for them. These visits also provide an excellent opportunity to network with industry professionals, and to experience company culture first-hand. ELITE also benefits participating companies, who can gain positive exposure among PhD trainees and identify talent for future hiring.

The concept of having trainees visit local companies and industries has previously been established as good practice at several other institutions. The industry site visit program developed by postdoctoral scholars at Massachusetts General Hospital in 2010 ( Abu-Yousif *et al.,* 2010), and the Postdoc Industry Exploration Program (PIEP) developed by postdocs at the University of California, Berkeley ( Nature, 2011; Tsang & Fisher, 2011) are examples of well-designed programs that directly expose postdocs to the type of research conducted and career paths available at a particular company, while at the same time giving them a glimpse of the company’s culture and providing networking opportunities. Since the inception of these innovative programs, a number of other institutions have followed suit to create their own, such as the Exploration Program for Industry Careers (EPIC) program at the Fred Hutchinson Cancer Research Center ( https://www.fredhutch.org/en/research/education-training/office-of-scientific-career-development.html); the Explore On Site (ExPOSE) program at the National Cancer Institute ( https://events.cancer.gov/cct/expose); the Bridges to Biotech multi-institutional partnership program in Maryland ( https://open.maryland.gov/blog/bridges-to-biotech-preparing-tomorrows-workforce-today/); the Industry Bridge Program at Scripps ( https://www.scripps.edu/about/campus-resources/working-at-scripps-research/campus-interest-groups/society-of-fellows/industry-bridge-program); and others. This sharp rise in new experiential site visit programs is a testament to the growing interest of training institutes and academic institutions in programs of this type.

Building directly upon the Massachusetts and Berkeley industry site visit program models, here we describe a variation on an industry site visit program that is a joint effort between three institutions: the NIEHS’ Office of Fellows’ Career Development (OFCD), the Duke University Office of Postdoctoral Services, and the Training Initiatives in Biomedical & Biological Sciences (TIBBS) at UNC. The unique three-institution ELITE site visit Consortium ( https://www.niehs.nih.gov/careers/research/fellows/involvement/committees/elite/index.cfm) was created to enhance the synergy from each of our three institutions’ pre-existing site visit programs and efforts, and was structured around the single-institution-based ELITE program that NIEHS initiated and formed in 2015.

Here, we provide a toolkit for running a joint multi-institutional industry site visit program. Our main aim is to remove barriers and administrative burdens of running such a program for other institutions by providing detailed standard operating procedures (SOPs), guidelines, and sample materials that other institutions could directly adapt and use. We also describe the preliminary career outcomes of ELITE consortium participants in an effort to determine whether the program impacted their career decisions and outcomes. Our purpose in providing a toolkit along with preliminary outcomes is to demonstrate initial program effectiveness and lessons learned in order to inspire development of experiential opportunities at other institutions.

## Program development

The ELITE program at NIEHS was originally modeled after successful programs in San Francisco, Boston, and Seattle. Briefly, attendees completed a biosketch and indicated interest in a site visit, the NIEHS Program Director selected attendees, attendees were required to attend a preparatory meeting, and NIEHS provided transportation to the site visit. UNC TIBBS also piloted a "Program for Industry Exploration" (PIE) prior to joining ELITE, and Duke was informally visiting companies about once per year. While these parallel institutional site visit programs were either modeled after others’ programs or developed in-house, we found that key adaptations were needed for continued success in our local environment. One of the most impactful adaptations involved inter-institutional collaboration. With this adaptation, all three institutions (NIEHS, UNC, & Duke) joined together to form the ELITE Consortium; we share the work of organizing visits, and an equal number of trainees from all three institutions may attend each site visit, regardless of the organizer that month. Besides decreasing the burden on any particular institution to plan all of the site visits, it enabled us to have a critical mass of attendees, which made better use of the company’s time, and thus increased their interest in hosting such an event since they could reach a more diverse audience—in short, because the program was explicitly a joint effort, companies were quicker to say “yes” to hosting a visit.

Another key adaptation involved the way in which trainees apply to attend a site visit. NIEHS began the original ELITE program in a manner similar to UC Berkeley’s Postdoc Industry Exploration Program ( Nature, 2011; Tsang & Fisher, 2011) which involved having those interested in the program submit a detailed biosketch to keep on file. The idea was that for each site visit, all biosketches would be reviewed and those with the closest match would be chosen to attend a visit. This biosketch model did not work well at NIEHS—likely due to its smaller postdoctoral population—and the decision was therefore made to switch to a new system. Interested attendees applied to attend each site visit by submitting a tailored cover letter as if they were applying to a job within the company. This adaptation has several benefits—1) it creates ‘up-front’ effort on the part of the trainee, thus selecting for those most interested and most likely to keep their commitment to attend a site visit; 2) it gives the trainee experience in writing a tailored cover letter; 3) it requires that the trainee conduct research on the company prior to attending; 4) companies may choose to receive these letters if they wish, and it could provide them with a potential talent pool. Institutions with related site visit programs ( Van Wart *et al.,* 2020) that involve extensive travel (e.g., Vanderbilt’s ASPIRE On the Road ( https://medschool.vanderbilt.edu/career-development/aspire-on-the-road/#:~:text=ASPIRE%20on%20the%20Road%20is,make%20well%2Dinformed%20career%20decisions) and the University of Chicago’s Trek Program ( https://careeradvancement.uchicago.edu/student-opportunities/treks) have also adopted cover letters as part of their application process.

Thus far, we have described some basics of the ELITE Consortium site visit program, as well as key adaptations that have made this joint program successful. Next, we describe the finer details of how the program works—including program variations at each member institution. Our goal is to provide a detailed framework that other institutions can use in order to seamlessly replicate, adapt, and/or expand this program for their trainees.

### Joint program development

Prior to beginning the site visit program in earnest, trainees were asked to provide input on the types of companies they would like to visit. Hence, pre-interest questionnaires were administered in some cases prior to establishment of the ELITE Consortium in order to gauge interest in site visits, help prioritize types of companies to visit, and to obtain a snapshot of career interests prior to ELITE program opportunities. A brief questionnaire was developed and employed (see representative sample results from one institution’s pre-program interest questionnaire, *Underlying Data S1* ( Collins *et al.,* 2020)). These results showed that the top three interests identified in this sample were to learn more about: research & development, pharmaceutical companies, and contract research organizations (CROs).

### Sample program description

**Figure 1.**
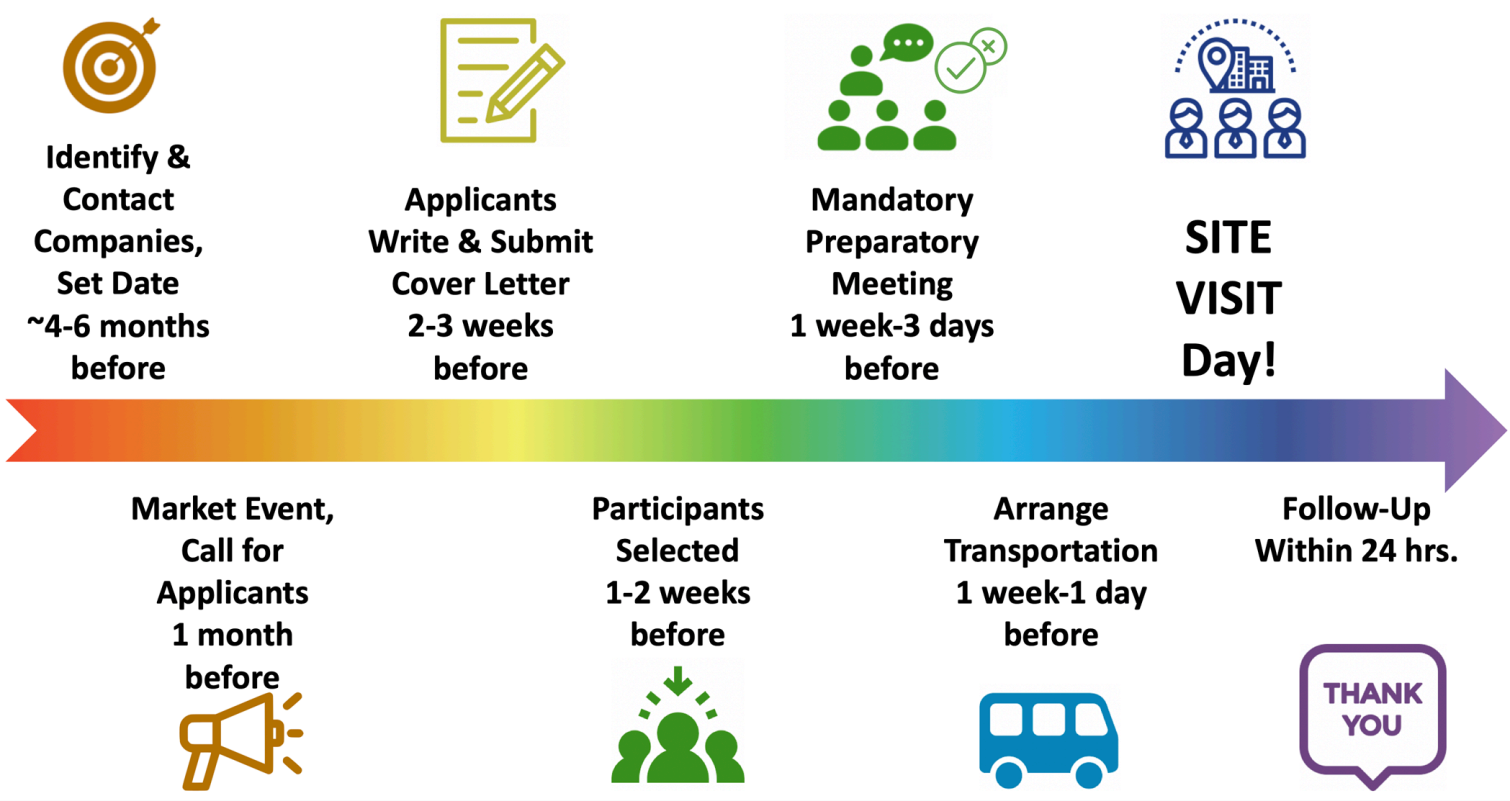
Chronological steps to organize an ELITE Consortium site visit. There are eight main steps involved in organizing an ELITE site visit, ranging all the way from identifying and contacting companies through to attending the site visit and following-up with the hosts.

***Steps.*** There are a number of steps involved in organizing a site visit (see Figure 1), ranging from identifying host companies all the way to follow-up. Here, we describe an overview of these steps; a detailed example Standard Operating Procedure (SOP) is also provided (see *Extended Data: S2* ( Collins *et al.,* 2020)) as a step-by-step guide that lays out many of the small details to consider when organizing a visit.

***Identifying and contacting companies.*** Based on the pre-program-development interest questionnaire, initial efforts were focused on attempting to organize site visits to company types that were of greatest interest, such as pharmaceutical companies and CROs. Cold-emailing company representatives met with some success (see *Extended Data: S3* ( Collins *et al.,* 2020)). Some connections were also made with potential host companies at conferences or events while networking or tabling (see example industry-geared flyer *Extended Data: S4* ( Collins *et al.,* 2020)) *.* In addition, we found that connecting to alumni working within a company was often a reliable way to gain initial buy-in for a company to host a visit. In our sample SOP (see *Extended Data: S3*), we provide a detailed framework for how to identify contacts within a company, and we recommend doing so approximately 4–6 months prior to an anticipated site visit date. Additional considerations include confidentiality and liability. While rare, companies have occasionally requested that attendees sign non-disclosure agreements; thus we suggest that organizers coordinate with the company and check with institutional subject matter experts (e.g. ethics officer, legal counsel) to ensure compliance with institutional guidelines. In case of questions from the company about liability, it is also important for institutions to clarify the extent of their coverage (e.g. liability coverage, workers’ compensation). Each of our institutions’ coverage extended to trainees while off campus and one should check their own institutions’ local policy.

**Figure 2.**
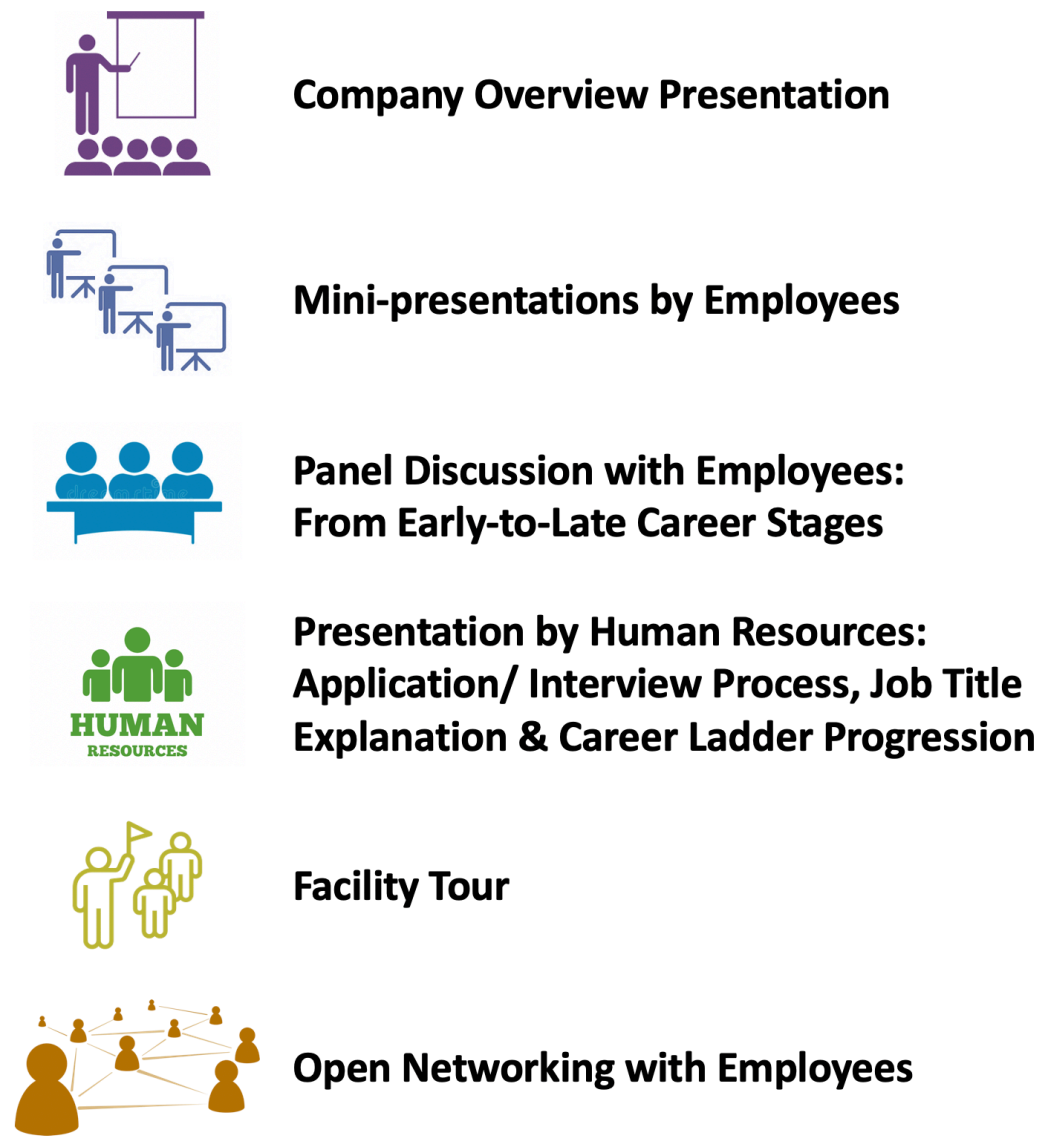
Examples of ELITE Consortium site visit activities. There are six key activities that may take place during a site visit, ranging from an overview of the company’s activities to networking with individual scientists at the company.

*Institutional variations:* NIEHS’ program was originally designed by trainees, and the organizing committee consists of staff within the Office of Fellows’ Career Development as well as a committee of trainees. For any particular site visit, an individual trainee can volunteer to be the lead organizer of a site visit; the SOP was originally written for trainees since their time at an institution is limited. The SOP helps to consolidate knowledge and best-practices, and it simplifies, streamlines and de-mystifies the process for trainee volunteers. Trainee volunteers also receive a sample ‘first contact’ letter template (see *Extended Data: S2*) to assist them in communicating with company representatives. Sample Agendas (see *Extended Data: 5* ( Collins *et al.,* 2020)) are also available as templates to share with companies in subsequent communications in order to help the company better understand what a site visit might entail (for common site visit activity ideas, see Figure 2). In lieu of trainee volunteers, institutional program staff at UNC and Duke within either UNC's Training Initiatives in Biomedical and Biological Sciences (TIBBS), or Duke’s Office of Postdoctoral Services serve as the lead organizers of site visits at their respective institutions, and they can utilize/adapt the SOPs for their institutions when planning a visit.

Regardless of the specific individual—whether trainee or program staff—organizing a given site visit, it is important to have some degree of institutional program support in order to provide continuity. Institutional program contacts typically convene annually to divide up the organizing load, and to determine which institution will organize a site visit for a given month. However, because organizing site visits can be an organic process, close communication between each institution is vital so that everyone is aware of potential site visits in the pipeline.

**Figure 3.**
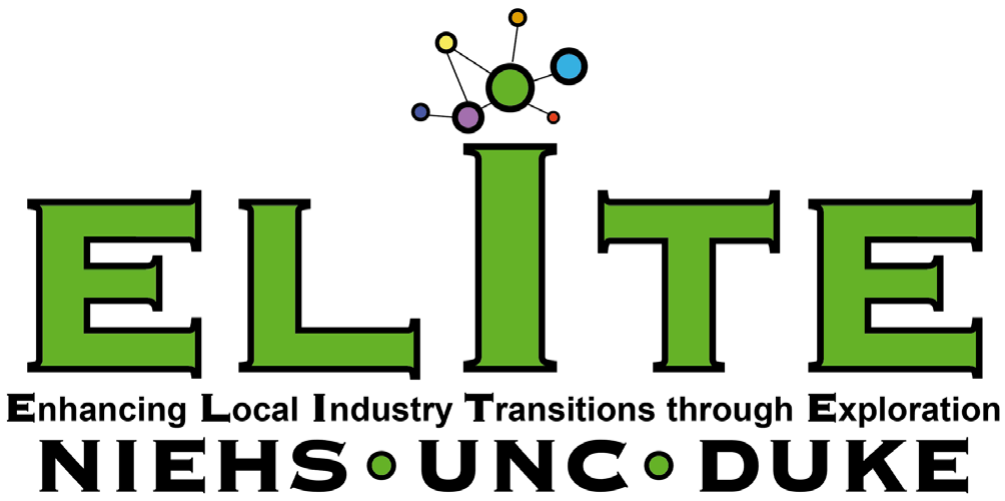
ELITE Consortium branding logo. We developed a unique logo for the program to ensure consistency in messaging, and to make the program easily recognizable when distributing marketing materials.

***Marketing/call for applicants.*** Once the lead organizer has identified a company and date for a site visit, the event is marketed at each institution via flyers and emails sent out to each institution’s trainee listserv (see Figure 3 for program branding logo). The lead organizer typically uses an SOP template for creating an email and/or flyer (for template email and flyer sample, *see Extended Data: S6 and S7* ( Collins *et al.,* 2020)) to market the event and call for applicants, who apply by writing a cover letter as if it were to be submitted to the company. In this initial announcement, we explicitly define the scope of the application and mention whether or not the company has requested to see the cover letters. In the vast majority of cases, the cover letter is not seen by the company, and is used solely for selecting site visit participants. The marketing materials are typically sent out approximately one month prior to the site visit, and they also include the date by which applications are due as well as the date of the mandatory preparatory meeting selectees are required to attend. Each institution may set their own application deadlines and preparatory meeting dates, and we collectively aim for application deadlines to be approximately two weeks prior to the site visit date.

***Applicants write and submit cover letter.*** Trainees apply for each site visit by writing a cover letter addressed to the company being visited. The letter must outline trainees’ background, fit, and interest in the company. As mentioned earlier, this gives trainees practice in writing cover letters; it ensures that they conduct research on the company ahead of time; it creates up-front ‘buy-in’ and increases chances that they will attend if selected; and it provides a potential talent pool for companies, as they are given the option to view the cover letters. However, many companies opt to not receive the letters, and they most often serve the intended purpose of preparing trainees. Trainees email their cover letter application to their respective institutional training office.

***Selection of participants.*** Participants are selected by institutional program directors; companies are not involved in the selection process. However, companies may view the cover letters if they request them during the initial planning phase. It is important to note for the institution with a trainee organizing committee (NIEHS), that trainees are not involved in the selection process and do not see the letters submitted. Before the selection process begins, institutions take note of the number of spots available for the site visit in question. Depending on what a company can accommodate, site visits typically include 15–50 participants, with the vast majority of visits hosting 20–25 participants. Keeping these numbers small allows for a smaller attendee-to-company-scientist ratio which could provide a more engaging learning and networking environment. The available spots are divided evenly among Duke, UNC, and NIEHS, regardless of the institution that is planning that particular site visit. As part of the selection process, cover letters are evaluated on the degree to which they are tailored to the company being visited. Typically, the number of cover letters submitted per institution allows us to accommodate all applicants by sharing open, extra spots across institutions if there are more applicants at one institution versus another. Once we begin to reach capacity, program directors make every attempt to accommodate all applicants—they often release their own designated spots to trainees, and they may contact the company host to ask whether the company can accommodate additional participants. In the rare instances in which capacity is reached, we may prioritize trainees who are more senior in their training fellowship (or nearing graduation). We may also prioritize attendance for those who have participated in the NIEHS ELITE organizing committee or those who have demonstrated other leadership involvement. Another point to note—since a trainee organizes the site visits at NIEHS, the organizer automatically reserves a spot to attend. 

After participants are formally notified of their selection, we may request that they each submit 2–3 questions they would like answered during the site visit. An example of questions submitted for one of the site visits is included (see example questions, *Extended Data: S8* ( Collins *et al.,* 2020)). These questions may be shared with the company ahead of time to help prepare their scientists for the types of questions that attendees may have. The purpose of this is to also encourage attendees to think more deeply about the company in advance, which will enhance their overall experience.

***Mandatory preparatory meeting.*** Once accepted to a site visit, trainees must attend a mandatory, though brief (30 minute), preparatory meeting (see sample preparatory meeting slides *, Extended Data: S9* ( Collins *et al.,* 2020)) that is organized and presented by each institutional program director. The preparatory meeting occurs anywhere from one week to a few days in advance of the site visit. At the preparatory meeting, program directors begin by discussing general site visit etiquette (e.g., networking tips, what to wear, and how to follow up with contacts using email or LinkedIn). In some instances, an institution may assist trainees in creating business cards and attendees are encouraged to exchange them during the site visit. After discussing general etiquette, directors present background research on the company found from sources including company annual reports, news articles, BioSpace, Google Patents, etc. Program directors then lead a discussion among trainees—many of whom have conducted their own research into the company. The meeting ends with a discussion about the site visit agenda and any confirmed company representatives that participants will meet. If any of the company representatives are institutional alumni, program directors try to point this out to participants in advance.

***Transportation.*** Transportation to a site visit varies by institution. NIEHS provides group transportation to all site visits. NIEHS identifies a location and time where participants gather well in advance of the site visit time. Institutional program directors reserve a van or multiple cars from the government motor pool. If a single vehicle can be taken that accommodates all participants, the institutional program director drives the participants to the site visit; if more than one vehicle is needed, a trainee volunteer may also drive. In rare situations, individuals may choose to drive their own personal vehicles depending on their personal circumstances. 

UNC has a mixed model in which participants may either ride in a van provided by the UNC program office for group transportation to site visits, or participants may instead choose to drive their personal vehicles and/or coordinate carpooling among themselves as needed. For those who opt to ride with the group (most of the participants), UNC identifies a location and time where participants gather well in advance of the site visit time. Trainees may volunteer to drive the program office’s van. For those who choose to drive individually or arrange carpooling among themselves, UNC instructs individuals to arrive to the site visit no later than 15 minutes before the site visit start time. 

Because Duke does not have institutional vehicles available to reserve, participants drive their personal vehicles and are encouraged to carpool. Carpooling is often discussed at the prep meeting. As at UNC, the option for personal vehicle use or carpooling is necessitated by the fact that many participants are spread out across large campuses. Trainees are encouraged to arrive at least 10 minutes early.

The fact that each of our three institutions have varied transportation models ranging from group-to-individual-transportation demonstrates that a site visit program can be successful with any of these models, and institutions should adopt the one that best suits trainees in their institutional environment.

***Site visit day.*** A site visit typically lasts three hours, and may involve a variety of activities (for sample agendas, see Figure 2 and *Extended Data: S5*), which differ based on the type of company visited—for example, whether bench- or office-based. A site visit frequently begins with a company overview presentation in order to provide participants with a basic understanding of what a company does. Site visits also often include a panel discussion with scientists working in a variety of positions across the company so that participants can learn about the different types of positions. Participants have found it informative to learn from both those in more senior roles as well as those who recently made the transition from academia to industry. Site visits may also include presentations from Human Resources representatives, and they may include an informal networking session to give participants a chance to mingle with company representatives. If the visit is to a company that primarily conducts bench research, participants are often provided a detailed tour of the company’s facilities.

***Follow-up.*** After a site visit, institutions follow-up in various ways. Participants are encouraged to immediately send an email thanking the company host and any company representatives that they connected with during the site visit. Participants are also encouraged to connect with company representatives on LinkedIn using a tailored invitation. Trainees may further follow-up via informational interviews with company representatives over coffee, lunch, or the phone. Some institutional program staff maintain a collection of thank-you cards; trainees are encouraged to sign and send a group snail-mail card to the company host. Some institutions may choose to send a gift basket or other token of appreciation to the company host (government funds are not used for these purposes). 

## Methods

### Data collection procedures

Once we organized the site visits, we identified key metrics to record each site visit in order to assess the program’s success. To maximize sample size, data from all three institution’s attendees were pooled and we standardized data collection criteria. Program participants’ career outcomes were evaluated by collecting publicly available career outcomes from the internet. 

Career outcome data were then grouped into taxonomic tiers jointly agreed upon by the authors, particularly those corresponding to Tier 1 of the commonly used PhD Career Outcomes Taxonomies ( Lenzi *et al.,* 2020; Stayart *et al.,* 2020; Xu *et al.,* 2018). Furthermore, due to the specific types of companies that hosted site visits, we further categorized each company into either Contract Service Organization (CSO), Biotech, or Pharma to reflect the Research Triangle Park’s primary markets. Some CSOs are also referred to as Contract Research Organizations or Clinical Research Associations, but for consistency as an umbrella term, CSO will be used hereafter. CSOs were identified by each company’s personal online description, as were biotechs and pharmaceutical companies. When biotech and pharma companies’ descriptions were confounded (for example, a Pharma Biotech company, or a Biotech company that made pharmaceuticals), we used an employee cutoff based on LinkedIn’s automatic company size categories. This was recorded in a common spreadsheet and then recoded for each participant’s career outcome.

Furthermore, attendance data was collected and collated for each site visit, and the number and type of site visits were coded by attendance and by whether a person was hired at the host company. Data was then coded to match the visit and career outcome on a number of criteria including: being hired at site visit company, being hired at a company of the same type (CSO, Biotech, or Pharma), and being hired at a company sharing the same Tier 1 coding (academia, for profit, government, etc.). MS Excel logic functions were used to create variables that quantified matching pairs (e.g., site visit job sector corresponding with hired job sector). We recorded if the participants attended a site visit, if they were hired at the visited company, if they were hired at a company of the same type (CSO, etc.), and if they were hired at a Tier 1 match (government, non-profit, etc.) by checkmark. We converted the Boolean checkmarks to binary 1’s and 0’s, and applied IF ELSE statements to the columns to simplify analysis. We compared the visited categories to the hired categories for each attendee. For example, IF attendee visit equals 1 (true), check the hire location to see if it matches the visit location and record 1 (true) for hired at site visit, ELSE record 0 (false). The same logic was applied to the type of company and tier one category of company. Due to not offering governmental site or teaching-intensive university site visits, the Sector (Tier 1) match analysis specifically compared For-Profit matches, but did not count other matches (Government, Non-Profit, Academic) because there were few site visits that fell into any of the latter three categories; hence only for-profit matches were included in the match count.

**Program summaries.** Site visits included in this summary were held between 2015-2019, including 30 total site visits with 24 unique sites/companies total ( https://www.niehs.nih.gov/careers/research/fellows/involvement/committees/elite/index.cfm contains full list), and 250 unique ELITE participants split approximately equally across the three institutions. We limited our career outcomes data analyses to those that graduated or were hired since attending an ELITE site visit (n=126; n = 47 Duke; n = 38 NIEHS; n = 41 UNC) through January 2020. Among those who participated and are now in their first position post-training, postdoctoral trainees (n=79) constituted 63% of the sample while doctoral students (n = 46) constituted the other 37%.

**Participants.** On average, ELITE participants who have since been hired into a first position attended 1–2 visits total (M=1.81, SD=1.38; min = 1, max =9; see Table 1 for attendance summary info) with 90% attending between 1–3 visits, which lasted approximately 3–4 hours per visit. Hence, our data suggests that positive benefits can arise with as little as 4–12 hours of time invested in professional development, networking, and experiential immersion during site visit activities.

**Table 1.** Number of site visits attended by ELITE participant alumni. The majority of participant alumni attended only one site visit (76, 60%). Some participants attended as many as seven (two individuals) or nine (one individual) ELITE site visits.

Number of visits	Frequency	Percent
1	76	60%
2	25	20%
3	12	10%
4	7	6%
5	3	2%
7	2	2%
9	1	<1%

**Companies.** Company sites visited included Contract Service Organizations (CSO; 46%, n=11), Biotech (25%, n=6), and Pharma (21%, n=5); a small percent were affiliated with academic institutions and did not clearly fit into any of these categories and were hence coded as “not applicable” N/A (8%, n=2).

**Planned analyses.** Summary statistics are provided to represent program participation for trainees and companies/organizations. Differences in career outcomes for graduate vs. postdoctoral trainees were tested using Chi-Squared analyses (e.g., company hiring match, sector hiring match). Logistic regression was used to test the hypothesis that attending more site visits was associated with a greater chance of being hired at a site visit company (or in that sector). IBM SPSS v26 was used to run analyses.

## Results

**Figure 4.**
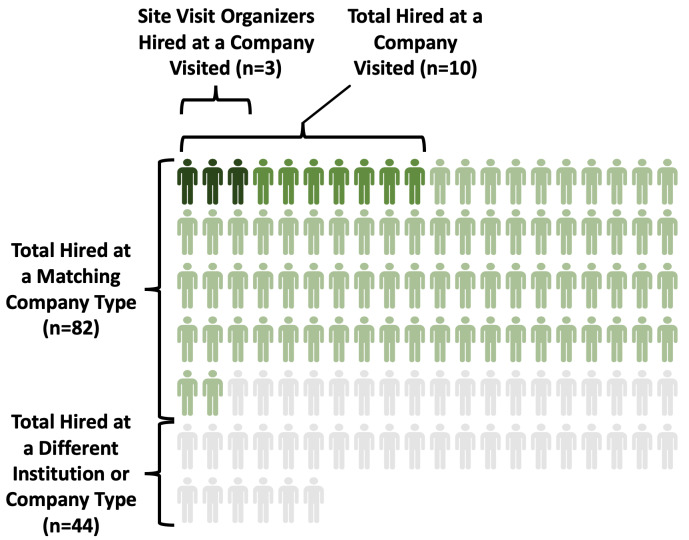
Depiction of where ELITE alumni were hired. The majority of ELITE attendee alumni were hired at a company type that matched those they visited (N=82, green icons (all shades)). Of those 82, ten were hired at the specific company visited, and 3 of those 10 were trainee site visit organizers.

Stated goals of the program included experiencing company culture, learning and practicing professional etiquette, and learning how to network and develop professional contacts. While getting hired at the company was explicitly *not* the stated goal of these site visits (and this is repeatedly emphasized to trainees in the mandatory prep visits), when hiring opportunities arose organically, they were a beneficial outcome for both the trainee and the company. While only 8% (n=10) were hired at a specific company they visited (see Figure 4), 65% (n=82) were hired at a type of company they visited (e.g., CSO) (see Figure 4). In addition, a majority of trainees (64%, n=81) were hired in the sector of a company they visited (e.g., for-profit). Thus, while site visits did not necessarily act as direct career pipelines, they did allow for attendees to gain a sense of the culture within a particular company type and sector, allowing for them to have a more confident match in their future careers.

A small number of trainees who organized a site visit had a much stronger likelihood of being hired at the company whose site visit they organized: 60% were hired by the company they organized the visit with (n=3/5). Furthermore, by definition, all of these were also company-type matches. This may indicate that an in-depth, meaningful interaction with company representatives while organizing site visits results in a stronger personal connection; it provides trainees an ability to demonstrate planning/organizational skills and professionalism; and it also offers an extended opportunity to connect and network beyond introductory levels. Furthermore, a person’s character and passion for the company may better shine through during prolonged planning interactions.

The large number (n=82) of participants hired into a similar company type (and even a few into the specific companies) suggests that attending site visits is helping the trainee find and/or display a good fit with the company, type, or industry. While we can’t isolate a single mechanism, it seems likely that trainees may have been able to confirm or deny their interest in a particular type and sector of company/organization, and perhaps these visits impact their interest and confidence in applying; knowledge of the roles and responsibilities of different positions; ability to create competitive application materials using appropriate industry terms; and networking connections within the industry. Further research is needed to tease apart the specific route(s) by which the site visits made a difference in trainee job searches and application processes.

Of ELITE participants hired at a matching company type, 57% (n=47) were hired at CSOs, 23% (n=19) at Biotech, and 20% (n=16) at Pharma. Participants from our sample were hired into CSOs at a high rate (at about twice the number compared with each of the other categories), which is not surprising given that the local job market is highly saturated with this type of organization. In fact, North Carolina has the greatest concentration of Contract Research Organizations in the world ( https://www.ncbiotech.org/about/history-of-biotechnology-north-carolina#panel6).

Overall, there were no differences in being hired into a specific company type when comparing postdoctoral and graduate trainees (Chi-squared test, X ^2^(1, N=126)=.26, *p*=.88). Although more postdocs were hired into positions across all three categories, there were more postdoc participants in the sample, so this did not differ from variations expected by chance based on the sampling distribution.

**Figure 5.**
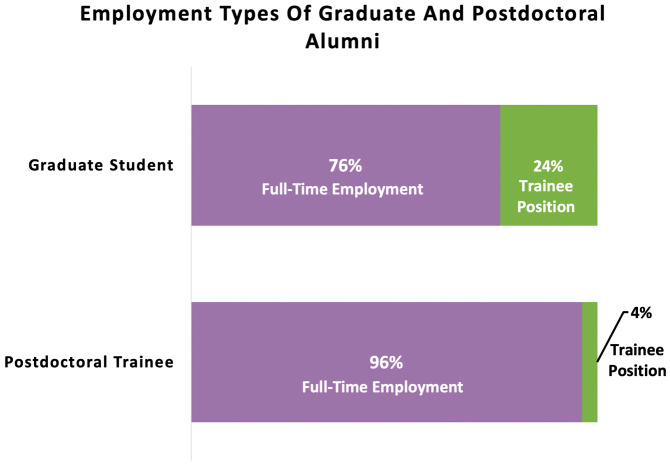
Percentages of graduate and postdoctoral ELITE participants hired into full-time employment vs. a trainee position. As expected, more graduate students entered into trainee positions as their next position (24%), whereas far fewer postdoctoral scholars entered into another training position (4%).

Tier 1 sector hires (For Profit as compared with Other – including Government, Non-Profit, or Academic) were compared between doctoral and postdoctoral trainees. For-profit vs. other (Chi-squared test, X ^2^(2,126)=10.29, *p*=.001) favored postdocs in for-profit positions, with a split roughly equally for graduate students in for-profit and other sectors compared with postdocs in other sectors (Chi-squared test, X ^2^(2,126)= 9.18, *p*=.002); however, the 2×2 Chi-Squared did not indicate a proportional difference from chance for the combined effect of postdoc vs. graduate student into for-profit vs. other sectors (X ^2^(1,126)= 1.90, *p*=0.17, NS). Hence, while a slightly greater proportion of postdocs entered for-profit directly compared with graduate students, the combined effect was not significant. This could partially be explained by the higher incidence of graduate students entering into postdoctoral training whereas a natural next step for a postdoc is full-time employment (see Figure 5).

To better understand if there was any effect for postdocs or graduate students transitioning into for-profit roles, we ran a further post-hoc analysis and removed trainees who entered into training positions as their next step (n=11 graduate students entered into postdoctoral training positions, and n=3 postdocs entered into further postdoctoral training in a new role or at another institution). When trainees who entered into a subsequent training role were removed from the sample, 67% of graduate students vs. 69% of postdoctoral trainees constituted matches on tier 1 sector for-profit hires (X ^2^(1, 112) = .03, *p*= 0.87, NS). Hence, any differences in postdoctoral versus graduate hiring rates seem to be accounted for by rates of continued training being more prevalent in graduate student populations than for postdoctoral trainees (which makes sense since they are chronologically further along in training by definition).

**Figure 6.**
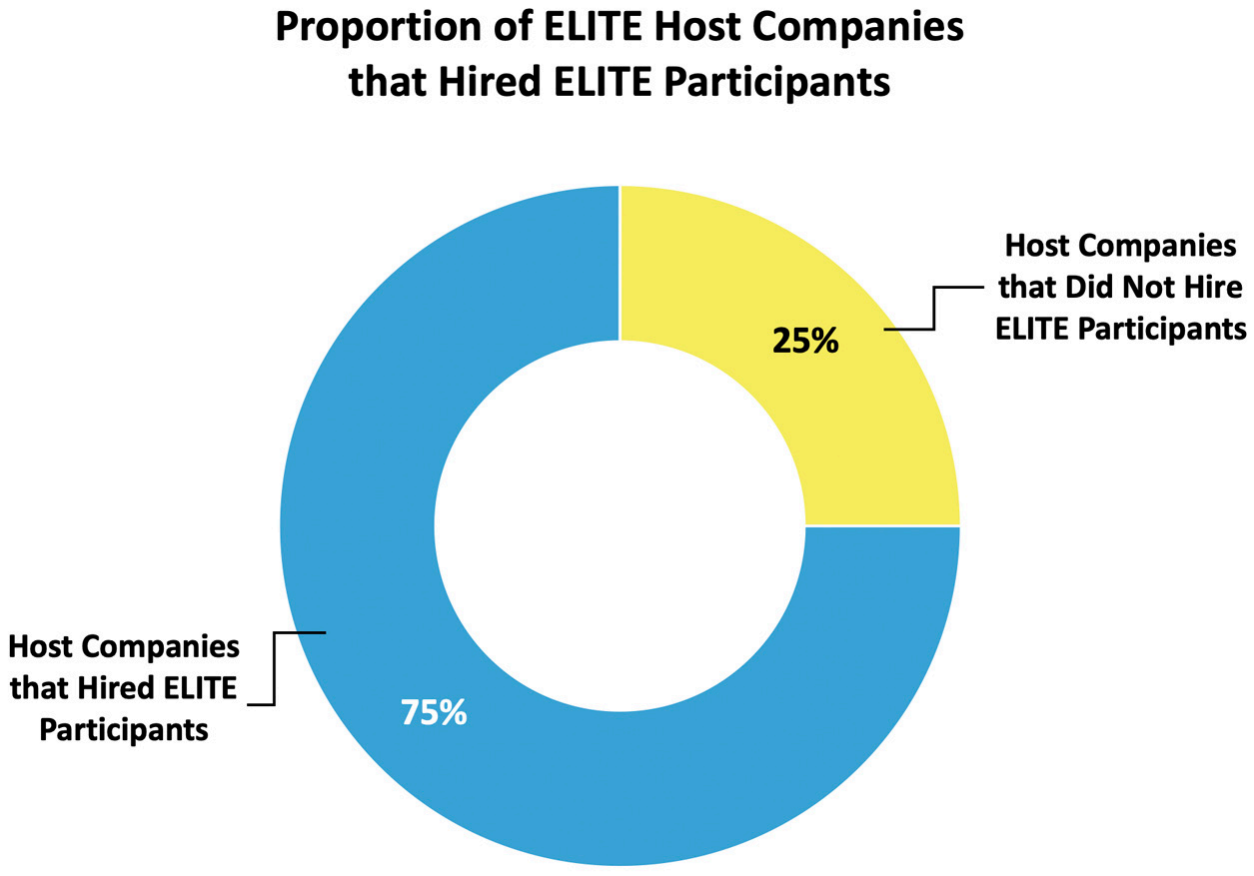
ELITE host company hiring percentages. A large majority (75%) of ELITE host companies have hired individuals who participated in the ELITE program.

In total, 75% (n=18/24, see Figure 6) of company partner organizations who have participated to date have hired a candidate who was an ELITE participant. The majority of companies have held one site visit to date, although a few have held more (in the current dataset, the site visit per company mode = 1, with 25% holding a second visit). Top hiring companies have hired up to 4 and 5 ELITE participants per company (range 0–5 hires, mode = 1 hire).

The number of site visits attended predict being hired at a company, with attendees’ hiring chances being about 1.5 times greater per site visit attended (OR = 1.58, p < .01, Wald = 6.90, B = .46, SEb= .17). Site visit participation continues to show a significant positive impact when controlling for graduate/postdoc status (OR = 1.53, p=.02); there was no significant difference between graduate or postdoctoral status (OR = .59, p=.54). In other words, attending more site visits made one more competitive as a future hire, regardless of whether one was a graduate student or postdoc.

**Figure 7.**
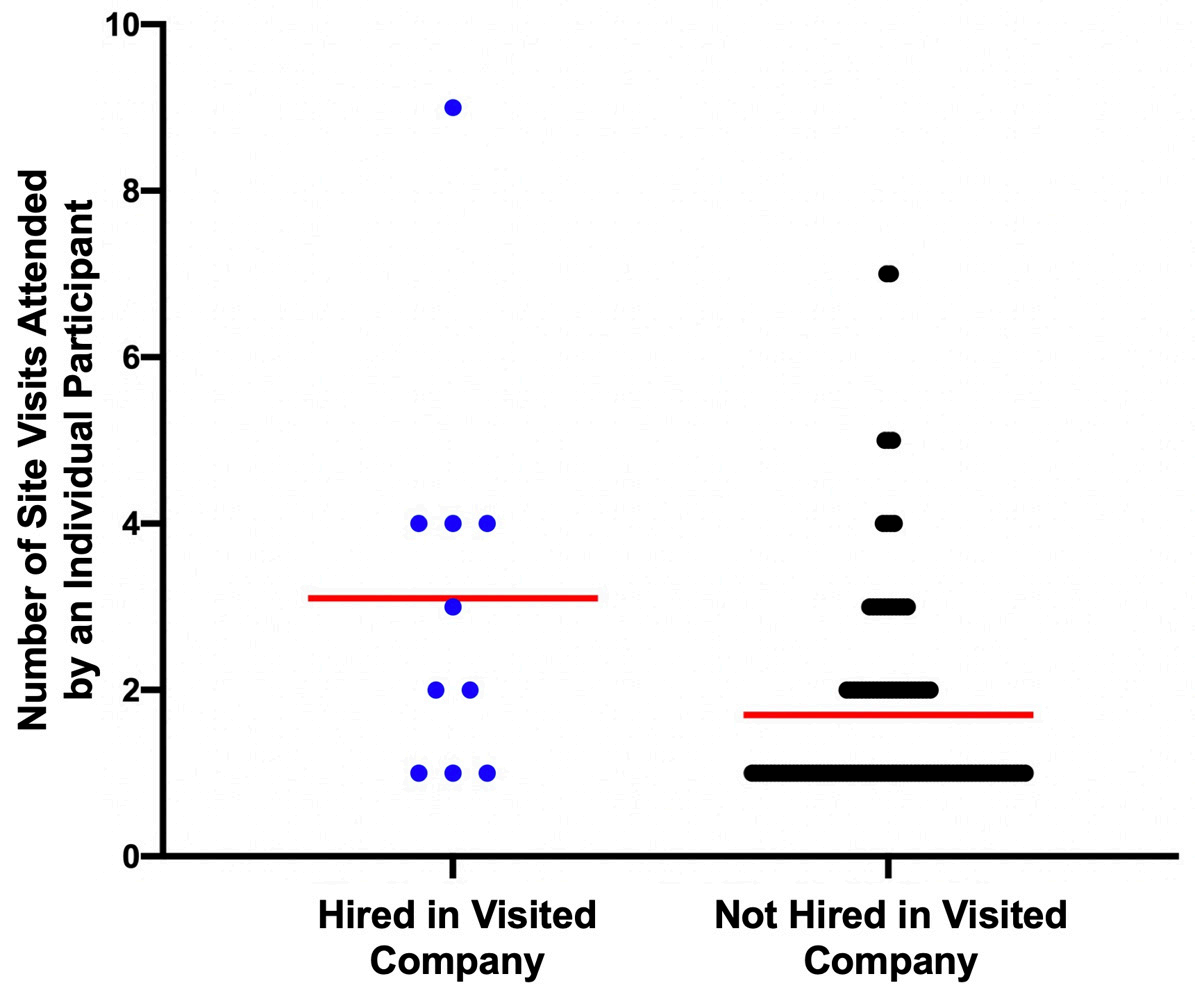
Individuals attending more site visits are more likely to be hired by an ELITE company visited. The ten individuals hired directly by the ELITE companies they visited attended more site visits overall on average. Those not hired into a visited company attended less visits on average (mean indicated by red bar).

Some of this effect could be driven by the fact that site visit organizers tended to attend more than others (see Figure 7); nonetheless we see a positive relationship between attendance and being hired at a similar company. When the three trainee organizers were removed from the analysis (10 minus 3; n=7 hired), attendance rates were no longer a significant predictor (OR = 1.16, p=0.58; graduate/postdoc remained non-significant, OR=.71, p=.69) of being hired, although the directionality of the effect was still positive. While this could be an artifact of lower power to detect an effect as the number hired at a company was previously already low (n=10), this suggests that being an organizer for a company site visit (which was also associated with attending more visits) may be a stronger predictor of later hiring success than simply attending.

## Discussion

Overall, the ELITE program was successful in its mission: to help trainees explore industry career options through site visits in Research Triangle Park, NC and to other employers beyond the traditional tenure-track. We saw significant numbers of both graduate students and postdocs hired at companies that match the type of company they visited, and several successful hires at the same sites visited. The visits were succinct and effective, even under the time constraints of a half-day visit. The program identified trainees looking for a variety of career options and companies looking for new talent, and coordinated the two fairly and representatively. Not all effects achieved significance which may be in part due to the limited sample size; nonetheless, emergent trends were identified.

In summary, there are different institutional models for a site visit program’s operation, and they can each work to meet the needs of the institution while still allowing for collaboration and serving to benefit the overall ELITE Consortium. It is important to figure out the optimal attendance capacity for visits and optimal frequency for each set of institutions and partners in your area, but it is also common to have trial and error while working out the ideal size.

The benefits and challenges of collaboration are varied. Benefits include less time per institution for planning; companies are more interested in hosting because they can reach multiple institutions simultaneously; and hosts have a guaranteed critical mass to make it worth the company’s time with higher attendance. Challenges include the additional complexity of coordination across multiple sites (requires more time, but happens less frequently per institution) such as establishing common date decisions, number of trainee acceptances, etc. While collaboration means there is the drawback of fewer overall spots per institution, a benefit is that when a particular host company is less popular at one institution, critical mass can still easily be reached. However, on occasion a host company may be popular across all three institutions, in which case less attendees per institution can be accommodated than if each had arranged separately. One other potential pitfall for institutions with trainees organizing the visits is that program leadership may need to educate trainee committee members on business etiquette in order to avoid any potentially negative interactions that could damage either the image of the Consortium or an individual’s professional image. To date, we have not experienced this pitfall, but it is a potential risk to consider – especially if early-stage trainees are involved in planning a site visit. In our experience, trainee involvement in planning has been a tremendous benefit, and having planners across sites has balanced-out responsibilities for planning well. However, it does require close interactions and real-time communication during the trainee site selection/ organization process, so working closely with colleagues across all institutions is important.

To better understand the time-associated benefits and/or challenges of collaborating with trainees and other institutions to organize a site visit, we estimated the amount of time it would take a program director to organize a site visit in different scenarios. Organized independently, it requires ~12.5 hours for a program director to organize a single site visit from start to finish—including all steps from identifying and contacting companies through attending the site visit itself and following-up afterwards. If a program director alone were to organize and attend one site visit per month for a year (12 visits/year), this would amount to an average of ~150 hours spent in a year. Collaborating with a trainee at one’s institution would reduce a program director’s hours by 20%. Extending this further to collaborate with two other institutions in the model described here reduces the total time spent per year to about 96 hours, which amounts to ~2.5 weeks of FTE time. These estimated hours all suppose that a program director is also attending each site visit, which takes up a significant portion of the time. If they choose not to attend the site visits, then the amount of time spent organizing in the collaborative model is further reduced to 36 hours per year. As illustrated, depending on the time constraints of the program director and needs of the institution, one can adjust their level of time commitment by using a program model that best fits their needs—whether by collaborating with more institutions and/or adjusting the number of site visits planned in a year.

### Participant benefits

We have also observed a multitude of benefits for trainees. For the institution with trainee-organized visits especially, the postdoc leadership experience, professional communication experience, and networking opportunities have provided valued benefits. In addition to our data that suggests benefits to trainees who may be future applicants in the for-profit sector or to a particular company type, we learned anecdotally that some trainees were hired specifically because they demonstrated outstanding communication skills while organizing an ELITE site visit on behalf of the Consortium. We surmise that the trainee/company interactions may allow company representatives to observe trainees in action, thus allowing employers to unofficially evaluate them as potential future colleagues. Other benefits include cover letter practice writing, learning about a variety of companies, and networking between postdocs and graduate students across all three institutions in the Consortium—besides the obvious benefit of networking with company professionals. This benefit was manifest in a number of ways, one of which included the fact that some company HR representatives voluntarily offered to prioritize ELITE participant job applications for review. Furthermore, a larger pool of program alumni from the Consortium has created contacts for future site visits, resulting in a synergistic effect which can then lead to future trainees being hired and/or learning more about company types through informational interviews, etc.

Anecdotally, some representative comments about participation benefits included the following:

“ *Thank you for selecting me as one of the applicants to attend the [Company] site visit. It was a great experience, and I enjoyed learning about the company culture and what it is like to work for a contract development/manufacturing organization. I think the ELITE program is a great way to help us postdocs make local industry contacts, and I hope to be able to participate in future site visits.*” – ELITE Participant

“ *The site visit to [Company] was awesome. Such exposure to industry is invaluable to Postdocs like me. Thanks for helping coordinate that visit*.” – ELITE Participant

“ *I love the environment of [the organization]. Thank you very much for your time and the previous chance of visiting [the organization]! Hopefully, I can naturally transfer to the clinical research field as you.*” – ELITE Participant

“ *The cover letter application is a good technique for applications and the actual site visit was excellent!*” – ELITE Participant

“[ *What I most liked about the [Company] visit was] the ability to hear from employees at varying stages of their career. The mix of hearing from early/newer employees all the way up to the VP and CEO was really great. Really enjoying the small group discussion/networking session opportunities to ask more questions!*” – ELITE Participant

Of ELITE participants questioned on the inaugural site visit (prior to the Consortium formation), 100% (n=4) indicated that the site visit met their expectations; other benefits were also mentioned—such as seeing what the company does, how the company works, and what it is like to work for them (Kristin Gabor, personal communication). A follow-up to one of the tri-institutional ELITE Consortium site visits indicated that 100% (n=12) thought it met their expectations and 100% (n=12) thought it was a positive use of their time. Though preliminary, these results suggest that trainees felt that they were getting what they wanted out of the site visit program; however, regular, comprehensive programmatic post-questionnaires would provide more robust evaluations of trainee satisfaction.

While we do not have conclusive data, it seems likely that at least some of the many trainees who did *not* match into a career outcome of a site they visited may have still gained perspective, experience, and information that impacted their decision-making in helping them make a confident choice away from those specific career paths. Data from one institution’s annual survey (n=82) indicated that trainees were both able to connect with other scientists and experience a career path. The survey also showed that ELITE Consortium visits helped trainees identify which career options were *not* a good fit (Layton, unpublished data). Trainees referred to ELITE site visits by name in an open text-response answer; this data was provided in response to the question, “In your time [here], have you changed your mind about the career path you plan to take?” (47 yes); “If YES, asked “Please explain the factors that contributed to your change in career interest.”

Furthermore, in response to the question: “Please list any suggestions for additional events or workshops that [we] should offer:” one respondent indicated that “[they] wish[ed] there were more options for ELITE site visits- the past few have seemed like they're all geared towards bench science careers in industry that are very specific to a certain area of research that I feel like only a few graduate students would be qualified for... So maybe something a little more generalized.” Hence, a future direction would be to create site visits to a broader range of companies, perhaps even outside the scope of science-specific companies.

Another possible benefit may be reducing the number of graduate students who continue for additional postdoctoral training versus moving directly to a permanent position after graduation. In one institution’s sample of ELITE participants (n=46 graduate students), only 24% (n=10) continued on to postdoctoral positions whereas 76% (n=36) continued to permanent employment. In comparison, national results from the NSF Survey of Earned Doctorates between 1998–2018 ( https://www.nsf.gov/statistics/srvydoctorates/#tabs-2), show that the percentage of life scientists that report entering postdoctoral training ranged from 59–65%. While this data is not conclusive, it at least suggests that there may be merit for further investigation. This is an issue that has long been a concern of the biomedical workforce ( NIH, 2012; NPA, 2012), and hence may provide a valuable tool to help trainees move into permanent positions more quickly and enhance the biomedical workforce with their talents.

### Employer benefits

In addition, we see evidence that employers found value in participating in the ELITE site visit as well, with some anecdotal unsolicited comments including:

“ *Thank you again for all of your work recruiting and connecting the [Institution] participants for the ELITE site visit on Tuesday. We enjoyed having you here and meeting your participants. I connected with a few [Institution] students including at the [Institution] Career Symposium and he has shown great interest in positions at [our company] so this was a great opportunity for him and others to come meet some of our R&D employees. Thanks again for taking the time to help set up this site visit and we look forward to continuing this partnership with [Institution]!*”- Employer

“ *It was really great to meet … the attendees. Everyone asked really wonderful questions and the energy during the networking was electric. So kudos to you guys for setting up such a successful program. We were happy to be a part of it!*” – Employer

“ *I wanted to let you know that one of the students you brought with you to the site visit interviewed with [our company] and we made an offer, which was accepted! This is a big win! We would love for you to use this to promote other students to apply to roles they are interested in and qualified for.*”- Employer

*“We really enjoyed hosting, and I always enjoy talking with folks about the company! I spoke with a number of attendees, and was very impressed by their questions about the technology and how we are using it. I hope to continue discussions with several of them, and we certainly look forward to hosting again in the future.*” - Employer

Beyond these unsolicited anecdotal comments, several employers have taken the initiative to invite the ELITE Consortium back on a yearly basis—even taking the lead in organizing subsequent site visits. In one particular case, an employer created a flyer with “ELITE Program Alumni” to showcase all of the hires they had made through this site visit program, complete with quotes by hires. Taken together, this suggests that companies and organizations that host ELITE site visits find some value in doing so. Nonetheless, a limitation of the current study is that it does not allow for granular data regarding host company benefits. Additional data should be systematically collected from companies during future site visit programs to explore what benefits are most impactful to host organizations, what features of the program convinced them to participate, and what data they would want to demonstrate return on investment of their company’s time and resources. This data could help inform program development to ensure that it provides mutual value and benefit to the hosts, organizers, and participants alike.

### Alumni engagement benefits

Alumni engagement is also a benefit, and we heard from alumni that reflecting back on their own career training and transitions from graduate or postdoctoral research, it was fulfilling to be part of setting up opportunities though ELITE for current trainees. Another benefit is that over time, site visit participants themselves may be hired at those or other companies and become the new points of contact to plan visits. This benefits the company to be able to tailor the visits to best create a PhD-trainee-centric experience. Furthermore, the alumni perspective allows the site visit host to better anticipate, plan for, and directly address common trainee questions.

“ *It was a pleasure to have you all visit yesterday. It was only a few short months ago that I was taking advantage of opportunities like this (thanks for those, by the way!) so I’m happy to be able to pay it forward now.*” - Alumni/Employer

### Industry/employer engagement benefits

Anecdotally, the ELITE program has resulted in additional employer engagement opportunities outside of site visits. In one example, a site visit resulted in a company representative volunteering to present a separate session at one of the ELITE Consortium member institutions, in which the representative explained the company’s career path options and trajectories to a broader audience. Fellows really appreciated the company representative explaining what the position titles meant, and explaining the career ladder progression at the company.

“ *It was great to see you here at [Company] and to interact with the fellows who came with you. I’m happy to help out as much as I can with answering questions and providing advice, so please feel free to keep using me as a resource for fellows who are interested in patent law. And if anyone sees something here at [our company] that they are interested in applying for, or if they want to speak with someone here about work in a particular field (immunotherapy, gene therapy, etc.), you can certainly reach out to me and I can put them in touch with the right person.*” - Employer

“ *It was a pleasure meeting all of you – there are so many opportunities available, whether you stay in academia or go to industry or government. Kudos for seeking out information to make the best decision for you (and extra kudos for the follow-up e-mail – it matters more than you might think). Happy to connect on LinkedIn, or chat further if you have additional questions.*” - Employer

## Limitations and future directions

A variety of limitations should be considered in the context of this study. One such limitation includes the potential for self-selection bias in that those individuals who apply to participate in this program may be more likely to already have career exploration experience and/or experience writing cover letters. We also recognize that preparation and understanding of career development processes may not be equitably accessed, which could influence site visit participation. Another potential limitation includes lack of a formal negative control comparison group. This is a common challenge in observational studies in which it may be unethical and/or impractical to deprive or delay participants’ access to professional development at crucial points in their training. Furthermore, in order to compare sector or company type matches, one would need an indicator, which in this case was attending an ELITE site visit in a particular sector/company type match. Hence there wasn’t an analogous ‘match/no match’ dataset for non-participants. Furthermore, intent to pursue additional training (e.g., postdoc) may influence immediate career decisions and could impact first-destination outcomes. The current study did not account for intent to pursue additional training and future studies should account for this intent versus intent to directly enter the workforce

Future directions could also involve exploring different binnings of company type as well as how interest, exposure, and first job match is related to specific roles (e.g., R &D). Our site visits covered a multitude of roles within each company type and hence career outcomes for specific roles could not be matched at this level of granularity. In addition, studies should explore other ways to define matches to include entrance surveys for whole populations assessing career interests which could be matched for participants versus non-participants at the end of their training. Furthermore, to complement quantitative analysis of career outcome matches, a comprehensive qualitative evaluation could provide more robust insight about the benefits of participating in the ELITE program. In addition, it will be important for future work to replicate this program using the toolkit at other institutions and evaluate its effectiveness, and/or to collaboratively conduct cross-site evaluations with similar existing programs.

Additional limitations included the lack of ability to examine demographic influences, unknown generalizability beyond life sciences, and reliance on a geographic convenience sample of companies. First, we were unable to examine career outcomes by demographics, such as gender, race/ethnicity, international status, etc. It will be important for these variables to be examined in future research in order to identify who is participating (or who isn’t, thus identifying potential barriers/inequities) as well as whether there are career outcome differences across groups. Second, our sample of trainees and companies were both life-science heavy by virtue of the population of participants served by the founding program directors and the high concentration of life science companies in this area. Third, we acknowledge that geographic location may influence concentration of available companies for site visits, especially given that Research Triangle Park is a major life science hub. However, future directions could explore virtual options as well as TREKs ( Van Wart *et al,*, 2020) for those institutions with fewer companies in close proximity.

Given the potential need for virtual visits now and into the future, we are currently exploring opportunities to pivot these types of programs into a virtual space, as it seems likely that this will be an area of future growth. In fact, leaders in graduate education have issued calls for a greater focus on preparing trainees for broader career options especially due to the implications of COVID-19 on a global economy ( Mathur, 2020). Answering these calls, we have initiated conversations with a company about hosting the first virtual site visit. It seems feasible that the company overview and panel discussions or mini-presentations may be the easiest to replicate in a virtual space. A virtual tour of the company may be feasible as well, since we learned that the company was already developing this capacity to host virtual tours; attendees may be able to view either a live or pre-recorded virtual company tour with the opportunity to ask questions at the end. While we also believe it is important to provide the opportunity for open networking, that may be an aspect of a site visit that is most challenging to replicate virtually; nonetheless, we are hopeful that the virtual networking options being explored will still provide an impactful and educational experience for attendees. Organizations such as the academic-industry collaborative non-profit organization University-Industry Demonstration Partnership ( UIDP) have proposed exploring virtual site visit best practices as well ( https://uidp.org/projects/; in the development phase of project at this writing). We believe virtual site visits will become increasingly pivotal for creating experiential learning opportunities for trainees interested in exploring industry, especially given the need for alternative options created by the global pandemic. Future directions should continue to explore the efficacy of virtual visits and other types of digital collaborations and connections between universities and companies.

## Conclusions

Our data suggests that a minimal amount of professional development time has shown significant positive impacts on career outcomes. Because this program has the potential to help many other graduate students and postdoctoral scholars, we wanted to reduce the barriers for other institutions to replicate such a program. Therefore, we have included a toolkit for developing a site visit program with local industry that can be adapted for single- or multi-institutional programs. The SOPs contain sample communications, marketing materials, policies, presentations, and program structure recommendations. We share lessons learned to create a robust, sustainable program. Furthermore, given resource scarcity, site visits can provide exceptionally effective professional development programming with minimal cost. In our experience, however, there is still a demonstrated necessity for including staff oversight and coordination time to develop a program that can run efficiently and consistently while ensuring professional business etiquette with industry – whether or not the staff take the role of the primary planners or as coordinators with a trainee planning committee.

## Ethical considerations

This activity was determined to constitute Non-Human Subjects Research (NHSR) as part of the NIH BEST Consortium under the auspices of the University of North Carolina IRB (IRB Number 14-0544). For the student surveys referenced herein, information sheets were provided to trainees, along with additional consent information for any who elected to complete surveys in which case they consented by continuing the voluntary survey.

## Data Availability

IRB approval for public data-sharing is limited to de-identified and aggregated data only, due to concerns of sharing personally identifiable information, which could be traced back to identify individuals included in the data set. Hence, personally-identifying information collected by individual institutions for their own alumni and used for coding purposes has been removed (job title, employer, LinkedIn profile or other job-related URL). Limited data-sharing for publication was approved by the respective IRBs as noted above. Open Science Framework: Creating and Sustaining Collaborative Multi-Institutional Industry Site Visit Programs: A Toolkit - Extended Data S1-S10, https://doi.org/10.17605/OSF.IO/RNSX3 ( Collins *et al.,* 2020). This project contains the following underlying data (information about each variable is embedded within the SPSS data files): Extended Data File – S1. UNC TIBBS Site Visit Interest Survey_DEID (SPSS) Extended Data File – S10. ELITE_DEID_coded (SPSS) Open Science Framework: Creating and Sustaining Collaborative Multi-Institutional Industry Site Visit Programs: A Toolkit - Extended Data S1-S10, https://doi.org/10.17605/OSF.IO/RNSX3 ( Collins *et al.,* 2020). This project contains the following extended data: Extended Data File – S2. ELITE Sample SOP Final Extended Data File – S3. ELITE First Contact Template Invitation Extended Data File – S4. ELITE Program Flyer for Industry Extended Data File – S5. ELITE Sample Agendas Extended Data File – S6. ELITE Template Email Announcement to Fellowslist Extended Data File – S7. ELITE Flyer Sample Extended Data File – S8. Sample Questions to ask Company at Site Visit Extended Data File – S9. ELITE Sample Prep Meeting Slides Data are available under the terms of the Creative Commons Zero "No rights reserved" data waiver  (CC0 1.0 Public domain dedication).
