# Supplementary material for: Creating and sustaining collaborative multi-institutional industry site visit programs: a toolkit
Source: F1000Res. 2022 Oct 28;9:1317. Originally published 2020 Nov 12. [Version 2] doi: 10.12688/f1000research.26598.2 (PMC7722534; doi:10.12688/f1000research.26598.2)
Supplement: Peer review history

# Peer review: Reviewer response for version 1

**DOI:** 10.5256/f1000research.29362.r74731

**Published:** 2020-12-07

**Authors:** Devavrat Vartak, Subu Subramanian

**Affiliations:** 1 Howard Hughes Medical Institute and the Department of Molecular and Cell Biology, University of California, Berkeley, USA; 2 Helen Wills Neuroscience Institute & School of Optometry, University of California, Berkely, USA

**Summary:**

 The article describes the conceptualization, implementation and preliminary evaluation of a career development program termed Enhancing Local Industry Transitions through Exploration (ELITE) that is aimed at postdoctoral researchers and senior graduate students in three research institutions (Duke University, NIEHS and UNC) in and around the Research Triangle Park, North Carolina. As the number of PhD holders keeps increasing while the number of tenure-track positions remains constant, there is an increasing need for trainees at research centers to explore industry opportunities. The ELITE program was established to facilitate site-visits to local industries that provide networking opportunities between the candidate and the company. Unlike other student/trainee driven career programs, at ELITE the institutional staff of the participating institutes serve as lead organizers. The authors provide 

 (i) a detailed toolkit, including standard operating procedures (SOP) for initiating first contact with company partners, selecting appropriate candidates from the application pool, organizing transportation and logistics for travel as well as fostering, and maintaining future relationships with the company partner organizations after the site visit. 

 (ii) an analysis of how successful the ELITE trainees are in finding jobs in the industry.

 The toolkit illustrates a framework with a detailed standard operating processes (SOP) for initiating first contact with company partners, selecting appropriate candidates from the application pool, organizing transportation and logistics for travel as well as fostering, and maintaining future relationships with the company partner organizations after the site visit. The toolkit and SOP are bound to be a useful resource for trainees at various research institutions wanting to organize site visits to local industries. 

 The framework is beneficial for all stake-holders, for the trainees, it provides a platform to learn writing cover-letters, business etiquette and conducting background research on companies before the site-visit. For the company partners it provides activity templates for conducting a successful site-visit. 

 The article reports that several of the trainees successfully transitioned to the industry of their choice and that many ex-alumni of the program have anecdotally praised it.

 In conclusion, considering the limited opportunities to find an academic position, the article supports an important point about the necessity of career development avenues for postdoctoral researchers.

**General Comments:**

**Minor comments:**

1. For the data collected for the manuscript, mention the years or months for which the ELITE program was operational, and comment on how representative the data is across the different years / institutions.
2. Figure 3 is unnecessary. 
3. Figure 4, 5 and 6 can be condensed to panels of a single figure, or a table.
4. Use “graduate students” in place of the colloquial “grad students”. Pages 10,13. 

**Minor clarification:**

1. If possible, is there data available on how many candidates of the ELITE program that successfully transitioned to the industry were international candidates? And how many industrial partners were willing to sponsor work visas?
2. Does the program only focus on life sciences related industries? Do other non-life science candidates benefit from the ELITE program?

Is the work clearly and accurately presented and does it cite the current literature?

Yes

If applicable, is the statistical analysis and its interpretation appropriate?

Yes

Are all the source data underlying the results available to ensure full reproducibility?

Yes

Is the study design appropriate and is the work technically sound?

Yes

Are the conclusions drawn adequately supported by the results?

Yes

Are sufficient details of methods and analysis provided to allow replication by others?

Yes

Reviewer Expertise:

Neuroscience

We confirm that we have read this submission and believe that we have an appropriate level of expertise to confirm that it is of an acceptable scientific standard.

# Response

**Published:** 2022-08-26

**Authors:** Tammy Collins

**Affiliations:** Burroughs Wellcome Fund, USA

**Response to 1: **Thank you for this suggestion, we have now included additional information to clarify this. Data includes all site visits from program inception (2015) through December 2019 and accepted job offers and/or start dates reflecting the same period. Excluding 2015, in which the program was just beginning to be formed, approximately 6 site visits per year were conducted. Participants were split approximately equally from all three institutions (Duke n=47; NIEHS n=38; UNC n=41). Please refer to the “Program summaries” section under “Methods” where we have modified the text to include this additional information.

**Response to 2:** Thank you for noting this; while Figure 3 doesn’t present data, it was provided as an example of program branding for institutions who may want to replicate their own site visits so we have chosen to retain it.

**Response to 3:** Thank you for pointing out that these figures could be consolidated. However, they were developed to help convey information through individual visualizations in order to highlight specific findings and better engage the reader. Because of the distinct purpose of each figure, we have elected to have them remain separate.

**Response to 4: **Thank you for pointing that out; we have modified the article and changed all incidents of “grad” to “graduate.”

**Response to minor clarification 1: **We acknowledge that this information would be especially informative for international students considering their career possibilities. While we do not have access to that data in the current sample, we agree that this is an interesting area to consider in future studies. We have now added a comment in the new limitations and future directions section: “Additional limitations included the inability to limit career outcomes by demographics, such as gender, race/ethnicity, international status, etc. It will be important for these variables to be examined in future research in order to identify who is participating as well as whether there are career outcome differences across groups.” 

**Response to minor clarification 2. **For the ELITE program, site visit participation was not explicitly limited to those in the life sciences, but we acknowledge that there is a strong representation of life science trainee participants based on the composition of those that program directors marketed the program to via listservs. Likewise, the ELITE program site visits were not strictly limited to life sciences by design--nonetheless, life sciences companies are very common in the Research Triangle Park area. We have added additional text within the new limitations and future directions section: “Second, our sample of trainees and companies were both life-science heavy by virtue of the population of participants served by the founding program directors and the high concentration of life science companies in this area.”

# Peer review: Reviewer response for version 1

**DOI:** 10.5256/f1000research.29362.r74733

**Published:** 2020-12-03

**Authors:** Michael Matrone, Linda Louie

**Affiliations:** 1 University of California, San Francisco, CA, USA; 2 University of California, San Francisco, San Francisco, CA, USA

In this article, the authors describe the development, implementation, and results of their “Enhancing Local Industry Transitions through Exploration (ELITE) Consortium,” a multi-institution industry site visit program designed to provide career exploration, company culture exposure, professional etiquette practice, and networking for biomedical graduate students and postdoctoral scholars in the Research Triangle region of North Carolina. They provide a detailed toolkit for other institutions to use in order to implement and execute their own site visit programs, and they discuss benefits for both the program participants and the employers visited. Additionally, the authors convey the career outcomes of program alumni in terms of their employment at contract research organizations, biotech companies, or pharmaceutical companies – the types of companies visited in this program. Specifically, the authors state that trainee site visit participation is positively correlated with being hired to the company visited, or to one of these three company types. In their cohort, this effect was significant for those who attended more than one site visit, namely trainees who organized the industry site visits, but not significant when these individuals were excluded from the analysis.

 The strength of this article is it provides a toolkit to establish this type of collaboration and program. The article, and the included toolkit, is a facilitators guide that includes timelines, standard operating procedures, communications, agendas, and data collection tools. Historically, a limitation of these types of programs is that their feasibility relies on geographic location – a university located in or near a pharmaceutical and biotechnology hub, like Research Triangle Park, will have a greater ability to form the partnerships required for such a program, simply due to proximity. However, as the authors note in their discussion of future directions, these programs can be adapted from a physical to a virtual space, utilizing technologies that are now more commonplace due to the COVID-19 pandemic, such as Zoom or Remo.

 Despite this strength, we are concerned that the two conclusions stated by the authors – that site visit participation is correlated positively with being hired at one of these company types, and that site visit participation helped trainees explore industry culture and careers – are not supported conclusively by the data provided in the article.

 Primarily, we feel it is overreaching for the authors to claim a causal relationship, significant or otherwise, between site visit participation and career outcome, given the data provided. We’re specifically concerned about the following: 

1. There is a lack of a control group for comparison. Inclusion of a local control, or comparison to national career outcome data for biomedical trainees, would help indicate whether the outcomes for this cohort differ from those who did not attend a site visit, or from national trends.
2. Selection biases and equity are not addressed. For example, is there a self-selection bias of program participants among trainees who may have already intended to apply for roles at a contract research organization, biotech company, or pharmaceutical company? Is the application process itself a barrier to entry for people in earlier stages of career exploration? Is there a selection bias for applicants who were more skilled at writing a mock cover letter, opposed to selection based on whether their intended goals aligned with those of the program? Since exclusion criteria are not detailed in the article, we wonder what criteria were used to evaluate how well a cover letter was tailored.

Secondarily, the authors include only anecdotal evidence for several claims pertaining to the success of their program. Statements, such as those below, seem overreaching given the limited data. 

- “…our data suggests that positive benefits can arise with as little as 4–12 hours of time invested in professional development, networking, and experiential immersion during site visit activities.”  
- “Thus, while site visits did not necessarily act as direct career pipelines, they did allow for attendees to gain a sense of the culture within a particular company type and sector, allowing for them to have a more confident match in their future careers.”  

These statements would be strengthened by inclusion of data pertaining to how these variables (time invested, understanding of company culture, etc.) were evaluated, and how the success of the program was evaluated overall. 

 To improve the authors’ conclusions with the data they have, we think a stronger case could be made by separating the program description, toolkit, and benefits from the story about career outcomes, allowing each to stand on their own. 

 For the program, toolkit and benefits story, addition of program evaluation criteria would benefit not only the toolkit for someone who wishes to emulate the program, but also strengthen the conclusions made about the benefits and impacts to the cohort studied here. Additionally, we suggest the authors investigate more the qualitative data from their program. It seems that they have quite a few written testimonials from both employers and participants; perhaps these responses could be coded, and further conclusions drawn that would flesh out the program benefits and could support the quantitative career outcomes data.  It would also allow for a more in-depth analysis of their program in context of the current literature. For example, how does the ELITE program compare with other research into internships, job simulations, and other kinds of career exploration activities, and can you draw some conclusions about how site visits provide some of the same benefits in a shorter amount of time? Or do they provide additional benefits, such as employer engagement, not offered by traditional career exploration activities? 

 For the career outcomes story, we feel that, aside from addressing our concerns stated above, studying a larger cohort is necessary in order to strengthen evidence that a positive correlation exists between site visit attendance and being hired at a company. To do so, the authors might consider partnering with another entity who runs a similar program. For example, the Society of Fellows, the postdoctoral association at The Scripps Research Institute, runs a long-standing industry site visit program, much like that at NIEHS. Not only would this increase the size of the cohort, but would also strengthen the authors’ most compelling evidence that being a site visit organizer significantly increases the likelihood of being hired in industry, as Scripps’ organizers are postdoctoral scholars.

Is the work clearly and accurately presented and does it cite the current literature?

Yes

If applicable, is the statistical analysis and its interpretation appropriate?

I cannot comment. A qualified statistician is required.

Are all the source data underlying the results available to ensure full reproducibility?

Yes

Is the study design appropriate and is the work technically sound?

Partly

Are the conclusions drawn adequately supported by the results?

Partly

Are sufficient details of methods and analysis provided to allow replication by others?

Yes

Reviewer Expertise:

career counseling, career development, program development and evaluation, career education research

We confirm that we have read this submission and believe that we have an appropriate level of expertise to confirm that it is of an acceptable scientific standard, however we have significant reservations, as outlined above.

# Response

**Published:** 2022-08-26

**Authors:** Tammy Collins

**Affiliations:** Burroughs Wellcome Fund, USA

**Response to 1:** We agree that this is a methodological limitation; more detail is addressed in our response to Reviewer 1Q4 who raised the same concern. Please note that we also address this concern in the newly added "Limitations and future directions" section of the manuscript.

**Response to 2:** Minimizing biases and ensuring equity during the selection process are important considerations for any program. We address a similar concern about selection criteria in our response to Reviewer 1Q1, and note that we also included discussion in the newly added "Limitations and future directions" section to address this. Equity and access issues have also been acknowledged and the following sentence has been added to the "Limitations and future directions" section of the manuscript: “We also recognize that preparation and understanding of career development processes may not be equitably accessed, which could influence site visit participation.” We also acknowledge that additional inequities exist (e.g., between Black job seekers’ networks and white job seekers’ networks). The ELITE program could help to explicitly overcome this by expanding networks and connecting to individuals primed to take action to assist in job seeking at the company.

**Response to 3:** Thank you for raising these issues. Reviewer 1 had the same concerns which we address in our response to R1Q4 and R1Q7. Additionally, we added text to the new Limitations and future directions section of the manuscript as indicated above. 

**Response to next statement about separating the manuscript: **The purpose of this study was to assess initial program outcomes and to provide a toolkit to help facilitate the adoption of site visits by other institutions. We feel that it is important to include initial program outcomes alongside the toolkit in order to provide an initial preview of the potential benefits of such a program. In the future, it will be interesting to conduct additional studies to better ascertain the mechanism(s) and the degree to which site visits may help trainees make more informed career decisions. We have modified text in the Introduction to include the following statement: “Our purpose in providing a toolkit along with preliminary outcomes is to demonstrate initial program effectiveness and lessons learned in order to inspire development of experiential opportunities at other institutions.” Furthermore, to acknowledge the importance of replicating the toolkit, we included the following statement in the new Limitations and Future Directions section: “It will be important for future work to replicate this program using the toolkit at other institutions and evaluate its effectiveness.” 

**Response to the next statement about evaluation: **We thank the reviewer for their feedback and agree that further research would allow us to better assess all of the benefits of conducting site visits. These will be important topics to pursue in future directions; we should note that all of the testimonials from employers resulted from unsolicited feedback and subsets of trainee self-report survey data (e.g., select cohort comparisons). We did not systematically collect qualitative data for a comprehensive evaluation and we agree this would provide a rich data source for future studies. The following statement has been added to the new Limitations and future directions section of the manuscript: “Furthermore, to complement quantitative analysis of career outcome matches, a comprehensive qualitative evaluation could provide more robust insight about the benefits of participating in the ELITE program.”

**Response to next statement about cohort size: **This is a fantastic idea and we agree it would provide not only a greater sample size but allow for additional multi-site comparisons of program success. Examples of great potential collaboration opportunities include but are not limited to the variety of program examples we included in the manuscript. We also modified the article to include Scripps’ Society of Fellows Industry Bridge Program. Thank you for bringing this to our attention. We also modified the text within the Limitations and future directions section to include the cross-site evaluation recommendation: “...to collaboratively conduct cross-site evaluations with similar existing programs.”

# Peer review: Reviewer response for version 1

**DOI:** 10.5256/f1000research.29362.r74735

**Published:** 2020-11-27

**Authors:** Nana Lee

**Affiliations:** 1 Department of Biochemistry and Immunology, University of Toronto, Toronto, ON, Canada

Methods were thoroughly written and a great idea for SOPs to involve multiple-institutional collaboration. Here are some points which should be addressed.

 1) Site visits are valuable, but the authors should address other variables which could have contributed to the hiring of these graduates, such as their professional development before or after these site visits. What other factors may have contributed to the hiring of these graduates? The authors should state these in their discussion  - as just having a site visit is not the only factor in the hiring of these graduates.

 a) Selection of attendees was by cover letters which already skews the pool of attendees who are deeply invested in their own career development, who had already perhaps attended cover letter-writing training.

 b) The selection process also used "leadership involvement" which selects for students who are driven in their career development already.

 2) Under joint program development: students were asked for their interests in R & D, pharma and CROs - some of these overlap, as all of these categories can contain R & D. This is related to point 5.

 3) Site visits - were there any liability waivers which the trainees had to sign? Just a question which could be a supplemental form in the future.

 4) Methods - data collection to "assess the program's success" - what defines success here? Career outcomes of participants? The proper negative control would be to collect employment data from a group of graduates who did not attend site visits, or even better, if the control group did not attend site visits, but had simliar interests, academic productivity and similar leadership experience. If this control was not collected, this has to be addressed in the discussion, as being a limitation of this study.

 5) The cutoff of 20,000 or less employees for biotech and 20,000+ for Pharma seems random - the type of career which the graduate enters should be assessed by their specific role. i.e. R & D, Medical Affairs, Business Development - regardless of the title/type of organization. Both biotech and pharma hold all of these roles. A more detailed analysis would be to see if the graduate obtained a job in the area of interest they had expressed prior/during site visit. Most students who are interested in R&D go into R&D, regardless if it is in biotech, pharma, non-profit government, CRO. This would affect the statement in the Results "Thus, while site visits did not necessarily act as direct career pipelines, they did allow for attendees to gain a sense of the culture within a particular company type and sector, allowing for them to have a more confident match in their future careers." If authors do not have this data (I could not tell from the supplemental information), this should be addressed in the discussion.

 6) Under program summaries, the authors state n=126 but the total number in figure 4 is 141.

 7) "Hence, our data suggests that positive benefits can arise with as little as 4–12 hours of time invested in professional development, networking, and experiential immersion during site visit activities." With the caveat that a negative control group does not exist here.

 8) The sample number of organizing trainees (n=5) is too small to make any conclusion. Although yes, the numbers who were hired by the company 3/5 is 60% - probably best to leave it as just the number and not a percentage.

 9) Fig 6: What are the numbers of graduates these companies hired who did not participate in the ELITE program? This would be a control group. If authors cannot obtain this data, this would be a discussion point.

 10)  "In one institution’s sample of ELITE participants (n=46 graduate students), only 24% (n=10) continued on to postdoctoral positions whereas 76% (n=36) continued to permanent employment." Trainees attending these site visits are more likely to be the ones who have decided to not pursue a postdoc. Maybe an intake question for the next site visit.

 11) Another factor which could have been discussed is geography. Research Triangle Park is a research hub - how many of these graduates are employed by these institutations because they live here? How many of these ELITE students left NC area with what type of career outcome?

 These points should be addressed in the discussion and/or future directions to make this publication a more thorough report and study.

Is the work clearly and accurately presented and does it cite the current literature?

Yes

If applicable, is the statistical analysis and its interpretation appropriate?

I cannot comment. A qualified statistician is required.

Are all the source data underlying the results available to ensure full reproducibility?

Yes

Is the study design appropriate and is the work technically sound?

Partly

Are the conclusions drawn adequately supported by the results?

Partly

Are sufficient details of methods and analysis provided to allow replication by others?

Yes

Reviewer Expertise:

Graduate professional development, industry collaborations, graduate curriculum, site visits, mentorshp, career outcomes of graduate students.

I confirm that I have read this submission and believe that I have an appropriate level of expertise to confirm that it is of an acceptable scientific standard, however I have significant reservations, as outlined above.

# Response

**Published:** 2022-08-26

**Authors:** Tammy Collins

**Affiliations:** Burroughs Wellcome Fund, USA

**Response to 1-a & b: **We made every effort to accommodate nearly all applicants for each site visit--including asking the companies if they could accommodate a few extra people when the applicant pool was high, and including giving up ‘program director’ seats to trainees. We acknowledge that writing a cover letter requires effort and planning, and at the same time we do not expect that this creates an insurmountable barrier for graduate and postdoctoral trainees. While we acknowledge that some self-selection bias may occur, the process of practicing cover-letter writing and thus researching the company will help strengthen their job application skills and thus competitiveness on the job market. In addition, we acknowledge that many factors may influence the hiring of graduates into these companies. Self-selection bias has now been acknowledged in a new limitations and future directions section added to the manuscript.

**Response to 2: **We agree that there is some overlap between R&D, pharma, and CRO; the pre-interest questionnaire allowed respondents to select from a wide variety of career categories and they could select interest in more than one. These distinctions were based on a convenience sample representative of the type of industries most common in our area, and we recognize that this could differ in other regions.

**Response to 3**: Regarding legal liabilities, such as if an accident were to happen--this is an important consideration for organizing a site visit. In our experience, trainees participating in an institutionally-organized site visit would typically be covered by the institution’s existing worker’s compensation policy. Site visit organizers should refer to their own institutions’ worker’s compensation policies to determine accident liability. In addition to accident liabilities, another common question that arises concerns confidentiality agreements. Companies occasionally requested that attendees sign confidentiality agreements stating that they would not divulge proprietary information learned during the visit. We have added the following text to the “Identifying and contacting companies” section: “Additional considerations include confidentiality and liability. While rare, companies have occasionally requested that attendees sign non-disclosure agreements; thus we suggest that organizers coordinate with the company and check with institutional subject matter experts (e.g. ethics officer, legal counsel) to ensure compliance with institutional guidelines. In case of questions from the company about liability, it is also important for institutions to clarify the extent of their coverage (e.g. liability coverage, workers’ compensation). Each of our institutions’ coverage extended to trainees while off campus and one should check their own institutions’ local policy.”  

**Response to 4: **The purpose of this study was to assess initial program outcomes and to provide a toolkit to help facilitate the adoption of site visits by other institutions. We agree that obtaining a negative control would be a cleaner test of the hypothesis. At the same time, we also acknowledge that there is always some element of self-selection bias in career development participation; hence, it is difficult to define a true negative control. One approach could be to compare these results to national or institutional career outcome averages, yet there are inherent difficulties in obtaining a representative sample that is truly comparable to a site visit participant comparison sample. Nonetheless, future studies could attempt to establish a baseline measurement of career outcomes pre- and post- introduction of site visit programs, while acknowledging that this method could also be confounded by economic and programmatic changes over time. This is beyond the scope of the current study but we agree that this is an interesting question which merits further attention across the career development field as a whole in identifying promising practices for effectively evaluating programs. We have acknowledged this limitation in the new limitations and future directions section of the manuscript.

**Response to 5:** Regarding company type, we searched the company website, LinkedIn, or the NC Biotechnology company directory to determine how to categorize a company based on their own self-identification. In rare cases where the self-identification was not made, company size was used as a factor in determining biotech versus pharma. The definition of these two has morphed over time, with size now being a commonly used factor toward distinguishing between the two (e.g., https://biotechhealth.com/biotech-vs-pharma/) and we used LinkedIn’s automatic company size designations as a cutoff which is now indicated in the manuscript.

 Regarding specific roles, thank you for pointing out that R & D may be a common career interest. It will be interesting to note for future analysis how company size and type factors into career choices relative to roles within a company. We also would like to point out the nuance that company cultures can vary quite a bit between large and small companies, and also between those that identify as contract research organizations relative to biotech or pharma, even across common R & D roles. We have acknowledged this in the new limitations and future direction section of the manuscript by adding the following text: “Future directions could look at different binnings of company type as well as how interest, exposure, and first job match is related to specific roles (e.g., R &D). Our site visits covered a multitude of roles within each company type and hence career outcomes for specific roles could not be matched at this level of granularity.” 

**Response to 6:** We understand that the figure may be confusing. The total number hired at a different company type (44) plus the number hired at a matching company type (82) is equal to 126. The number hired at a specific company visited (10, of which 3 were site visit organizers) is still part of the 82 hired at a company type; extra description was added to clarify the relative proportion of the 82 company type matches that involved being hired at a specific company visited. To clarify these distinctions, we have modified the figure legend. The following text modified to say the following: “The majority of ELITE attendee alumni were hired at a company type that matched those they visited (N=82, green icons (all shades)). Of those 82, ten were hired at the specific company visited, and 3 of those 10 were trainee site visit organizers.”

**Response to 7:** Thank you for pointing this out. We have addressed this in our response to point 4 above. 

**Response to 8:** We agree that results should be interpreted with caution because in some cases the “N” is small. Because of that, we chose to include both the “N”s as well as the percentages so that it would be informative for the reader and also allow for comparisons of differing proportions in order to highlight patterns.

**Response to 9: **This is a great area for future discussion and outside the scope of the current study. We have added a paragraph on limitations and future directions in which we address the need for a control group, including non-participant career outcomes (second paragraph). We acknowledge that this is an important area of future consideration.

**Response to 10: **We agree that this is a key area of consideration, and it will be important to examine whether site visits are simply selecting for those who otherwise would not do a postdoc, or whether they are facilitating quicker transitions into permanent employment. It will be interesting to further pursue these questions and adjust our intake questionnaires accordingly in future studies. The following has been added to limitations and future directions section: “Furthermore, intent to pursue additional training (e.g., postdoc) may influence immediate career decisions and could impact first-destination outcomes. The current study did not account for intent to pursue additional training and future studies should account for this intent versus intent to directly enter the workforce.”

**Response to 11:** A large percentage of ELITE attendees are employed in NC; as more institutions are reporting their career outcomes, it is becoming apparent that geography indeed plays a role--increasing evidence demonstrates a strong correlation between the location in which someone trained and the location in which they became employed. For instance, the top job location for all ELITE institutions with publicly available location information was North Carolina (e.g., Xu et al 2018, https://www.niehs.nih.gov/careers/research/fellows/alumni-outcomes/index.cfm, https://bbsp.unc.edu/professional-development/career-outcomes/, https://gradschool.duke.edu/about/statistics/all-departments-phd-career-outcomes-statistics, https://postdoc.duke.edu/statistics-coalition-next-generation-life-science-0 The correlation between training and subsequent close proximity of employment is further supported when examining outcomes of graduate students and postdocs around the United States (Mathur et al 2016). Examples of collated institutional career outcome information can be found by exploring the Graduate Career Consortium’s database of member institution outcomes ( https://osf.io/28dn6/), or the Coalition of Next Generation of Life Sciences’ member institution outcomes ( https://nglscoalition.org/). The following has been added to the article in the Limitations and Future Directions Section: “Third, we acknowledge that geographic location may influence concentration of available companies for site visits, especially given that Research Triangle Park is a major life science hub. However, future directions could explore virtual options as well as TREKs (Van Wart et al, 2020) for those institutions with fewer companies in close proximity.” 

**Response to last statement: **We have addressed these points by adding a Limitations section in the manuscript and have expanded upon the Future Directions section. 

 Thank you for thoughtful feedback; we believe that this has significantly strengthened the manuscript.
